# The emergent role of small-bodied herbivores in pre-empting phase shifts on degraded coral reefs

**DOI:** 10.1038/srep39670

**Published:** 2017-01-05

**Authors:** Caitlin D. Kuempel, Andrew H. Altieri

**Affiliations:** 1 Smithsonian Tropical Research Institute, Apartado 0843-03092, Balboa, Ancon, Republic of Panama; 2 Marine Science Center, Northeastern University, 430 Nahant Road, Nahant, Massachusetts 01908 USA; 3 Centre for Biodiversity and Conservation Science, School of Biological Sciences, University of Queensland, St. Lucia, QLD 4072, Australia

## Abstract

Natural and anthropogenic stressors can cause phase shifts from coral-dominated to algal-dominated states. In the Caribbean, over-fishing of large herbivorous fish and disease among the long-spined urchin, *Diadema,* have facilitated algal growth on degraded reefs. We found that diminutive species of urchin and parrotfish, which escaped die-offs and fishing pressure, can achieve abundances comparable to total herbivore biomass on healthier, protected reefs, and exert sufficient grazing function to pre-empt macroalgal dominance following mass coral mortality. Grazing was highest on the most degraded reefs, and was driven by small herbivores that made up >93% of the average herbivore biomass (per m^2^). We suggest that previously marginal species can achieve a degree of functional redundancy, and that their compensatory herbivory may play an important role in ecosystem resilience. Management strategies should consider the potential role of these additional herbivore functional groups in safeguarding natural controls of algal growth in times of increased uncertainty for the world’s reefs.

Ecological and anthropogenic stressors have altered biodiversity and ecosystem functioning at a global scale and are expected to intensify in coming years^1^,^2^,^3^. The ability for ecosystems to compensate for loss in biodiversity affects their resilience to stress and is largely dependent on how tolerances are distributed within and among functional groups^4^. Determining the role of functional groups and their response to stressors is critical in making predictions about ecological trajectories and in prioritizing conservation efforts. Coral reefs, among the world’s most diverse and valuable ecosystems, provide an ideal model to better understand ecosystem responses to a changing environment and the role of functional groups in promoting resilience.

Phase shifts from coral to macroalgal dominated states have emerged as a significant threat to coral reef resilience^5^,^6^,^7^,^8^. In the Caribbean, coral reefs have experienced a marked decline in live coral cover in recent decades due to the cumulative impacts of stressors including hurricanes, disease outbreaks, and thermal bleaching events^9^,^10^,^11^ which freed space for competitive colonizers such as macroalgae^12^. The likelihood of transitioning between states is thought to be mediated by the strength of top-down control in the form of herbivory^5^,^13^. Understanding the factors that influence transitions between reef states and the ability of coral reefs to recover from disturbance is essential to inform management and conservation efforts across these valuable ecosystems.

While there has been considerable debate as to whether algae can overtake and displace coral, it is generally accepted that algae can opportunistically occupy dead coral reef substrate and prevent recruitment and recovery of coral communities in disturbed areas^6^,^14^,^15^. The shift from coral to algal dominance, as described in Jamaica^13^, has been a crucial model for understanding potential trajectories of disturbed reefs and how they may relate to consumer dynamics. However, recent evidence suggests that coral-algal phase shifts may be less consistent than previously assumed^16^. Uncertainty in the frequency and occurrence of phase shifts questions our mechanistic understanding of coral reef dynamics and our ability to predict coral resilience and recovery^11^,^17^,^18^,^19^.

Caribbean coral reefs exhibits high levels of algal dominance relative to other tropical regions^16^,^18^. This has been attributed to severe anthropogenic disturbance, loss of key, large-bodied herbivores, and coral degradation through factors such as disease and storm surges^8^,^13^,^16^. We used the recent mass mortality of coral associated with a hypoxic event on the Caribbean coast of Panama, in which >90% of coral died on some reefs, to test the generality of coral-algal phase shifts following a major disturbance. This region is subject to pervasive anthropogenic stressors that are representative of much of the Caribbean and other human-dominated coral reef ecosystems including: coastal development, eutrophication, overfishing of large herbivorous fish^20^,^21^,^22^, and the epizootic die-off of the dominant, herbivorous urchin *Diadema antillarum* that was first reported in Panama^23^. These factors create conditions that increase the susceptibility of algal dominance following coral mortality.

Here, we show that top-down control can pre-empt phase shifts on degraded coral reefs where diminutive members of the herbivore assemblage reach sufficient levels of biomass and grazing pressure to prevent algal dominance following coral mortality. We evaluated the relationships between herbivore populations, coral and macroalgal cover, and herbivory pressure using a series of field surveys and herbivore manipulations. In addition, we conducted caging and algal transplant experiments to test the importance of grazing relative to other factors that can limit algal growth, and to partition the effects of different herbivore body-size guilds. Our findings suggest that even in an anthropogenically modified system with diminished populations of some consumers, previously marginal species can play an important role in ecosystem resilience by compensating for the loss of top-down control by larger-bodied herbivores.

## Results

### Habitat and Herbivore Surveys

**Figure 1:**
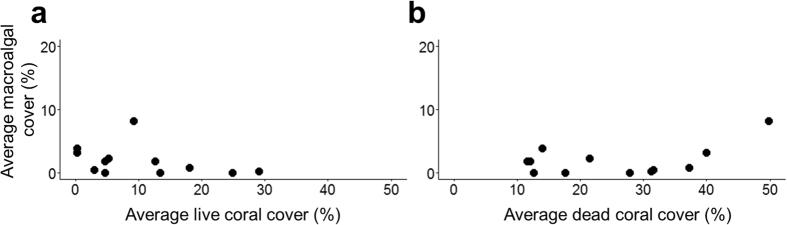
Associations between dominant benthic covers on coral reefs. (**a**) Relationship between average percent of macroalgal cover and average percent of live coral cover (n = 12) (**b**) Relationship between average percent of macroalgal cover and average percent of dead coral cover (n = 12) on surveyed reefs in the Bocas del Toro Archipelago.

Habitat surveys revealed very little macroalgae in the Bocas del Toro Archipelago, regardless of live or dead coral cover. Percent cover of macroalgae was low across all 12 sites, with an average of 2.58 ± 0.8%, while live coral cover varied more widely, averaging 10.4 ± 2.7%. Dead coral, an indication of reef degradation and substrate availability for algal colonization, was widespread, averaging 25.57 ± 3.7% across all sites. There was no relationship between macroalgal cover and live coral cover (r^2^ = 0.18, p = 0.17, Fig. 1a) or macroalgal cover and dead coral cover (r^2^ = 0.11, p = 0.29, Fig. 1b).

**Figure 2:**
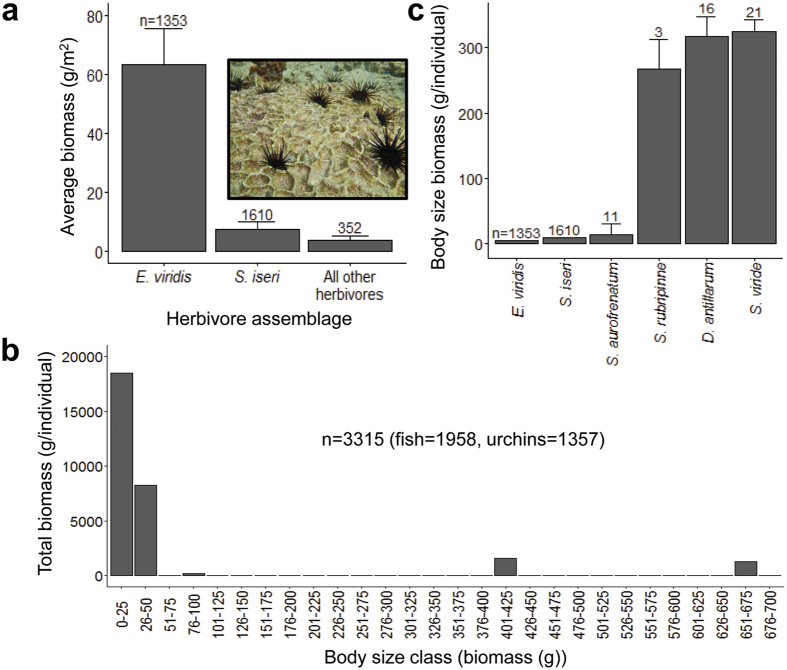
Distribution of total herbivore biomass across body-size classes and dominant herbivore species (n = 3315). (**a**) Average (±SEM) biomass of the diminutive urchin (*E. viridis*) and striped parrotfish (*S. iseri*) versus all other herbivores with inset photograph from STRI Point study site depicting typical *E. viridis* activity on a dead coral head during the day (Photo credit: C.D.K.), (**b**) average relative body size (±SEM) comparison of herbivorous parrotfish and urchins found at our study sites and (**c**) total biomass pooled across sites within each body-size class. The sample size (n) is denoted in each figure, respectively.

Herbivore identity and abundance showed consistent patterns across all sites. The dominant herbivores were the urchin *Echinometra viridis* and parrotfish *Scarus iseri. E. viridis* accounted for 99.7% of all urchins (n = 1357, with only two *Diadema antillarum* and two *Lytechinus* spp.) and *S. iseri* accounted for 82.2% of all herbivorous fish. Across all sites *E. viridis* biomass averaged 63.5 ± 12 g/m^2^ while *S. iseri*, averaged 7.6 ± 2.2 g/m^2^. Together these small-bodied species averaged 71.1 ± 12.4 g/m^2^, or 93.7% of the average observed herbivore biomass per m^2^ (Fig. 2a). Additional surveys revealed that these two species ranked smallest in body size relative to other herbivorous parrotfish and urchins in our study system, with individual body sizes over an order of magnitude smaller in terms of biomass (Fig. 2b). *E. viridis* and *S. iseri* comprised 80.9% of total herbivore biomass within the smallest body size class (0–25 g), which contained the majority of the total biomass observed, while *S. iseri* comprised 98.4% of the next smallest and second most prominent size class (26–50 g) (Fig. 2c).

### Algal Transplant Experiment

**Figure 3:**
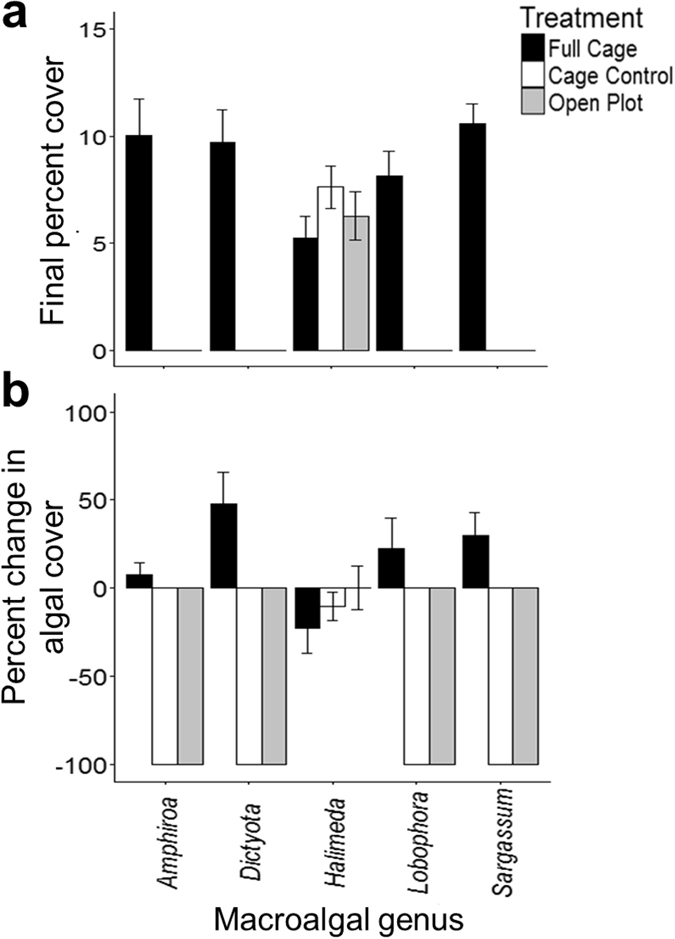
Response of five genera of macroalgae to herbivory (n = 30). (**a**) Average (±SEM) final percent cover as a function of caging treatment and (**b**) average (±SEM) change in percent cover as a function of caging treatment and macroalgal genus during the 22-day transplant experiment.

In our macroalgal transplant experiment, herbivory had an overall negative effect on algal biomass (F_2,108_ = 32.62, p < 0.0001) and the intensity of herbivory differed between algal species (F_4,108_ = 87.72, p < 0.0001). Four of the five algal species were completely consumed within the first week of the experiment in plots exposed to herbivory (Fig. 3a), and exhibited 7.5–47.9% increases in percent cover in plots that excluded herbivores (Fig. 3b). Only the well-defended *Halimeda* spp. had appreciable biomass remaining in open plots at the end of the trial period (Tukey’s HSD, p < 0.05, Fig. 3a) and experienced decreases in percent cover in the herbivore exclusion treatment (Fig. 3b).

### Herbivory x Nutrient Enrichment Experiment

**Figure 4:**
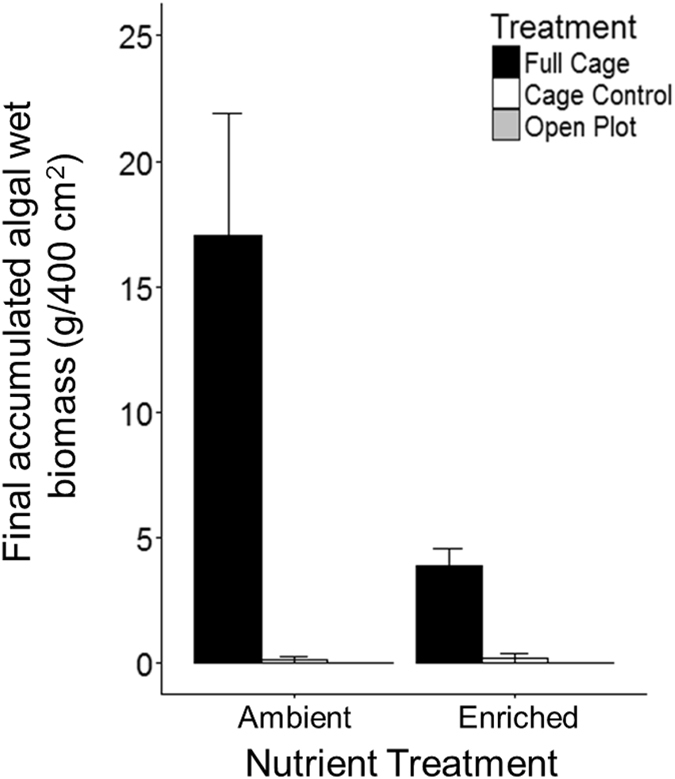
Mean algal biomass (±SEM) on experimental tiles under factorial nutrient enrichment (ambient, enriched) and herbivore exclusion (full cage, cage control, open) treatments (n = 44).

Herbivory and nutrient enrichment had an interactive effect on algal biomass (F_2,38_ = 8.90, p = 0.0007, Fig. 4). Exclusion of herbivores led to 20-fold higher algal biomass relative to controls. Within the herbivore exclusion treatment, algal biomass was higher in the ambient than enriched nutrient plots (Tukey’s HSD, p < 0.05).

### Herbivore Size Exclusion Experiment

**Figure 5:**
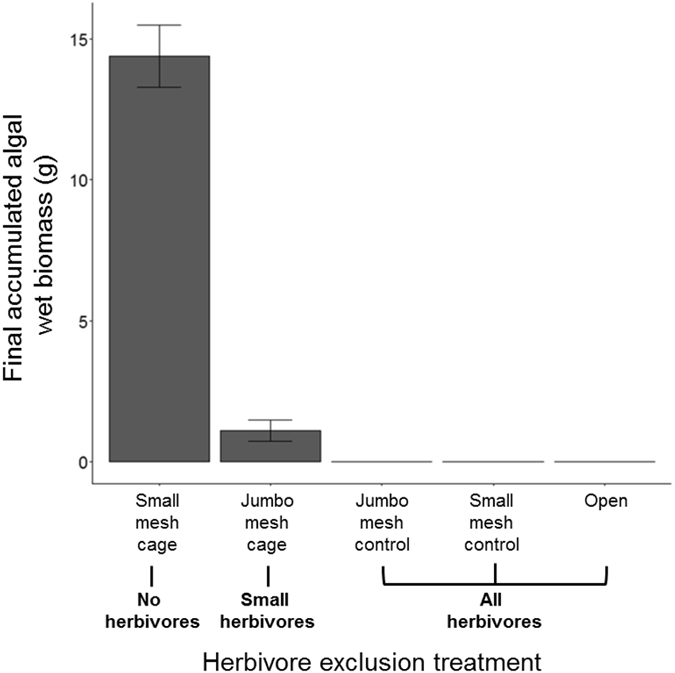
Grazing impacts as a function of herbivore body size. Final mean algal biomass (±SEM) as a function of caging treatment (n = 48): jumbo mesh excluded large herbivores but allowed access for small herbivores including the reef urchin *E. viridis* and striped parrot fish *S. iseri*, small mesh excluded all herbivorous fish and urchin, and the jumbo mesh control, small mesh control, and open treatments allowed access to all herbivores.

Herbivory had a significant effect on algal biomass (F_4,47_ = 237.44, p < 0.0001). Significantly less algal biomass remained in the jumbo mesh cages that permitted access by small herbivores, but excluded larger herbivores, than in the small mesh cages that excluded all herbivores (Tukey’s HSD, p < 0.05). The small herbivores that were able to access algae in jumbo mesh cages consumed >90% of available algae before the end of the experiment (Fig. 5).

### Multisite Herbivory Assay

**Figure 6:**
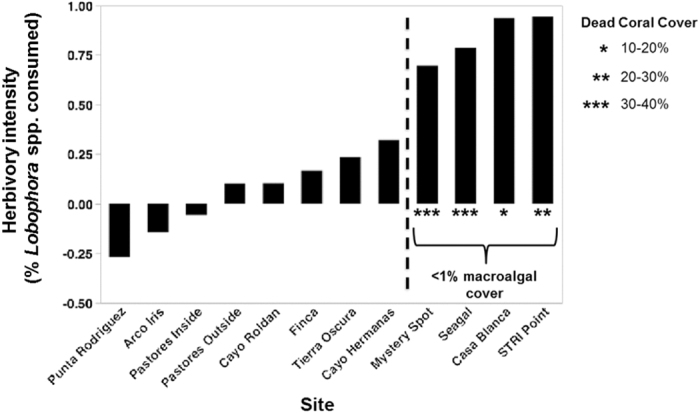
Herbivory intensity[(Mean caged biomass-Mean open biomass)/Mean caged biomass] by site, depicting sites with significant herbivory (≥50% of algae present was consumed; right of dashed line) and sites with non-significant herbivory (left of dashed line; n = 12).

Herbivory rates differed by site (F_11,213_ = 23.79, p < 0.0001, LS Means Contrasts: p < 0.05, Fig. 6), with four of twelve sites showing significant effects of herbivory after the two-day experiment. These four sites were among the lowest for average algal cover (all with <1% cover) and highest for average dead coral substrate (all with >12% cover). Our surveys revealed less fleshy macroalgae at sites with significant herbivory intensity (≥50% of *Lobophora* spp. eaten) compared to sites without significant herbivory (t = 2.61, p = 0.032). We did not detect a relationship between herbivorous fish biomass and the percent change in *Lobophora* spp. (r^2^ = 0.003, p = 0.87), whereas increases in urchin biomass were positively correlated with the percent change in *Lobophora* spp. in our algal transplant (r^2^ = 0.36, p = 0.038) across sites.

## Discussion

Our results confirm that a macroalgal phase shift has not occurred in the Bocas del Toro region following a mass coral mortality event. High cover of dead coral, low cover of macroalgae, and a lack of correlation between the two are contrary to expectations of an algal phase shift^16^. Furthermore, herbivory was found to be sufficiently strong to prevent macroalgal dominance in this ecosystem with a history of strong fishing pressure and a skewed consumer community. Species of small herbivores, which have likely escaped such pressures, had high cumulative biomass levels and significant impacts on algal biomass within surveys and size exclusion experiments. Herbivory assays revealed that grazing pressure was strongest at the most degraded reefs, and was negatively correlated with the abundance of macroalgae across all sites. These findings indicate that small-bodied species can achieve sufficient grazing function to compensate for the loss of keystone grazers and highlight the importance of initial diversity within the herbivore community in preventing phase-shifts and associated hysteresis on coral reefs.

Three key aspects of our experimental results support the conclusion that herbivory pressure is strong enough to control macroalgal growth in our system. First, the cover and biomass of all but one alga was significantly reduced by herbivores in our algal transplant experiment. The one alga species not affected, *Halimeda* spp., has strong structural and chemical defenses against most herbivores^24^ explaining why it alone persisted. Slight decreases in the abundance of *Halimeda* in the caged treatment may have been due to secondary effects of transplantation, or to the presence of a small *Halimeda-*specialist herbivore, such as the sea slug *Elysia luca*^25^, which would itself have benefited from the protection of cages. Second, there was a positive response of algal recruitment and growth when herbivory was limited by caging or territorial damselfish in our herbivory x nutrient experiment. Rapid growth of algae only in the caged plots of our algal transplant experiment further indicates that herbivory is a primary factor controlling algal dynamics. Third, algal biomass on caged tiles was higher in ambient nutrient plots than enriched plots. Similar results, which initially seem contrary to expected nutrient-algal dynamics, have been observed in other coral reef studies^26^,^27^,^28^,^29^,^30^. Biomass may have been higher in ambient plots due to a stimulation of grazing by mesograzers (e.g., small snails, urchin recruits, amphipods that were small enough to enter cages between maintenance visits) due to higher food quality in fertilized plots. The role and relative abundance of nutrients in the Caribbean is still unclear^18^,^31^, but Bocas del Toro is known to have episodes of eutrophication due to freshwater run-off^32^, making nutrient limitation unlikely.

As further indirect evidence for the importance of herbivory, our experiments found no support for four alternative factors that are commonly known to limit algal growth. First, physical conditions at our most degraded site (STRI Point) were adequate for algal survivorship and rapid growth for the duration of our three-week algal transplant experiment and four-month herbivory x nutrient experiment. For example, all algae that were significantly reduced in open plots showed growth in caged treatments of up to ∼47% increase in cover during the 22 day transplant experiment. These rates are consistent with macroalgal growth in other studies that excluded herbivores over similar time periods^18^, despite algae in our experiment being constrained to the substrate of dead coral fragments on which they were transplanted. Moreover, growth occurred regardless of potential shading by roofs in cages, suggesting that light is not the primary factor limiting algal abundance. Second, less algae was found in nutrient enriched caged plots relative to ambient caged plots in our herbivory x nutrient experiment indicating an absence of nutrient limitation. Third, we observed recruitment and increases in algal biomass within ambient caged plots and plots that were defended by territorial damselfish (and subsequently excluded from analysis) in our herbivory x nutrient addition experiment. This suggests that algal propagules are available and can settle and survive in our study area. Finally, the lack of correlation between dead coral cover and macroalgae cover, coupled with the high cover of dead coral substrate across sites (an average of 25.6 ± 3.7% of all benthic substrates surveyed), eliminated substrate availability as a plausible control of algal abundance.

Herbivore surveys revealed sufficient consumer abundances to produce the strong grazing effects that we observed. The biomass of small-bodied *S.iseri* (individuals <50 g) on these reefs rivals the total parrotfish biomass in some areas of the Bahamas, Belize, Turks and Caicos, Cuba, and Puerto Rico^11^,^33^, and combined with the urchin biomass, is nearly 2.5 times greater than other areas of the Caribbean^34^*. Echinometra viridis* escaped the disease that decimated *Diadema* populations^23^, while *S. iseri* is diminutive (mature at ~65 mm^35^) and therefore not a targeted fisheries species. Results of our size-selective exclusions provided experimental evidence for the important role of these diminutive herbivore species as nearly all macroalgae were consumed in jumbo mesh cages, which permitted herbivory by only grazers within this small size class.

Current reports and recommendations focus on the role of grazing by *Diadema* and large parrotfish in combating macroalgal phase shifts^11^,^36^. Recently, Edwards *et al*.^37^ noted that relatively higher numbers of smaller-bodied fish accounted for the difference in biomass, but not abundance, of herbivores on fished reefs across the Caribbean and hypothesized subsequent declines in herbivory potential. However, our results indicate that, in at least some instances, secondary species of grazers can rise to perform functions similar to large grazers, and thereby play an important role in system stability. This may be especially true in degraded, overfished, and heavily impacted ecosystems, as in our Caribbean study area. In the Indo-Pacific, the relatively rapid recovery of reefs following disturbance has been attributed to robust herbivore communities^5^,^19^, but our results are the first, to our knowledge, to suggest a similar potential due to functional redundancy in the herbivore community of the Caribbean. As aspects of our study system vary relative to some other well-studied areas of the Caribbean, which experience higher wave exposure and water flow, it will be important to monitor impacts of small bodied herbivores to determine the ecological contexts where similar dynamics can and do occur. Transitions to small-bodied herbivore communities will likely become more prevalent as populations of previously marginal herbivore species increase in response to predator loss and reductions in dominant competitors due to disease and anthropogenic impacts such as overfishing, which impacts nearly 70% of Caribbean reefs^38^.

The importance of small herbivores could be an indicator that phase-shifts observed on Caribbean coral reefs in the decades following *Diadema* loss were a transitory state that occurs until other herbivore species rise in prominence and are able to functionally compensate. The rise of *Diadema* is thought to be driven by the loss of predators and competing herbivores^39^; therefore the loss of *Diadema* can be expected to have impacts on herbivore composition. We hypothesize that there may have been a demographic lag in compensatory grazing following the loss of *Diadema*, and now, 30 years later, *E. viridis* has reached a biomass threshold which can compensate for the functional extinction of *Diadema.* Perhaps, not coincidentally, we first observed this transition and compensatory grazing on the Caribbean coast of Panama where the epizootic wave of *Diadema* mortality originated^23^. However, the timeframe of this apparent shift is still unclear and should be considered in future work.

Studies conducted 10–15 years following the disease outbreak showed that other urchin species (including *E.viridis*) failed to increase in biomass^40^, or to produce the same grazing intensity as *Diadema*^41^, which was largely attributed to predation pressure. Overfishing in Bocas del Toro is likely to have diminished populations of predators of herbivores, allowing for unconstrained grazing to reach sufficient levels to suppress macroalgal growth. Our common observance of urchins in open, vulnerable positions on emergent surfaces during both day and night (inset photo Fig. 2a) support this idea, which is consistent with a release of smaller-bodied herbivores through a trophic cascade as hypothesized in other coral reef ecosystems^36^,^42^, and thought to occur more generally in systems where apex predators have been lost^43^. It is also possible that populations of *E. viridis* and *S. iseri* have responded positively through bottom-up donor-control^44^ to an increase in primary production fuelled by availability of dead coral substrate for algal growth. For example, in Moorea, Adam *et al*.^45^ found a similar increase in small parrotfish populations following a crown-of-thorns starfish outbreak that significantly increased open substrate for grazing, which they attributed to prior food limitation^45^. High grazing rates and rapid turnover of productivity to grazer biomass would explain why standing stock algal biomass has remained low despite this potentially elevated primary productivity and may lead to continued population increases among herbivores in coming years.

Other studies conducted in the Caribbean of Panama have also remarked on the high abundance of *E.viridis*^20^,^46^, whose crucial role was further supported by the positive correlation we observed between herbivory intensity and *E.viridis* biomass. While large *E. viridis* populations can reduce algal abundance and maintain open substrate for potential coral recruitment, the impacts on coral reef recovery and resilience of this species in the Caribbean have yet to be explicitly tested. Aronson *et al*.^47^ hypothesized that historical populations of *E. viridis* in the Bocas del Toro region may have facilitated transitions from one dominant coral species to another by controlling macroalgal growth. However, studies of a similar scenario off of the coast of Kenya found that both fish and urchins were equally effective in removing algae, but increases in the abundance of another *Echinometra* species led to decreases in live coral cover, topographic complexity, and eventually losses in fisheries productivity due to erosion and the removal of coral recruits after settlement^48^,^49^. Further work should identify the impact of *E. viridis* on coral recruitment and substrate erosion in the Caribbean.

We observed spatial variation in the intensity of herbivory pressure, which suggests the potential for important feedbacks related to phase-shifts and resilience in coral reefs. Our 12-site herbivory assay revealed that herbivory was strongest at the sites with both the lowest macroalgal abundance and the highest coverage of dead coral. This contrasts with previous studies where significant herbivory was documented on reefs with low live coral cover, but high macroalgal cover ranging from 20–50%^33^. The ability of herbivores to limit macroalgal cover to 0–8.6% across our study sites is a novel finding in a Caribbean system and questions the idea that herbivores can only exclude algae on high live coral-cover reefs^50^. Furthermore, our finding of strong grazing coupled with high levels of bare, dead coral substrate is in contrast to the idea of an “upper limit” of bare substrate at which grazing becomes insufficient^33^,^51^. Perhaps this is because dead coral substrate is an attractive habitat for *E. viridis*^52^. Our observation that herbivory was especially intense at sites with low abundance of algae and high cover of bare dead coral substrate is further evidence for feedbacks that could prevent the system from shifting from a coral to algal phase as previously hypothesized^53^.

We found that diminutive herbivores that escape common environmental and anthropogenic pressures can reach sufficient biomass thresholds to pre-empt phase shifts to macroalgal dominated states. At such densities, these grazers appear functionally redundant to other species, including large parrotfish and *Diadema*, that are recognized as the first defence against algal overgrowth on Caribbean reefs^11^,^36^. Future research should explore the conditions and timeframes that determine whether compensatory herbivory by species of small herbivores occurs, whether it is sufficiently strong to reverse phase shift trajectories back to a coral dominated state, and the implications for coral recruitment and resilience. Determining the respective roles of invertebrate and fish herbivores, particularly in highly fished areas, will give further insight into mechanisms controlling algal phase shifts, herbivore populations, and factors influencing variation in grazing intensity and complementarity.

Large herbivores provide important reef functions, however we suggest that management and monitoring efforts aimed at preventing phase-shifts from coral to macroalgae on reefs should broaden to include the role and importance of diminutive and apparently marginal species of herbivores, as stress induced shifts in size structure may impact ecosystems functioning more subtly than the loss of entire functional groups^4^. This may be particularly important during disease outbreaks or other scenarios where the replenishment of large herbivore communities is difficult or impossible to control through management actions. Furthermore, consideration should be given to potential lags and implications of compensatory grazing on reef health, recovery, and resilience. Maintaining functional redundancy and diverse coral reef species assemblages will help to safeguard natural controls of algal growth in times of increased uncertainty for the future of the world’s reefs.

## Materials and Methods

### Study sites

The Bahia de Almirante is a semi-enclosed lagoon in Bocas del Toro, Panama (see Supplementary Fig. 1) which has historically supported well-developed coral reefs^54^, but has been heavily impacted by terrestrial development and overfishing^22^. In 2010 there was a mass coral mortality event associated with hypoxia which created large areas of bare, dead coral substrate available for macroalgal growth (A. Altieri *unpublished data*). These conditions created a unique opportunity to test processes controlling algal dynamics. We conducted studies from July-November 2013 on 12 fringing reefs throughout the lagoon, which are dominated by lettuce coral (*Agaricia* spp.) but varied in the cover of dead coral (lacking any visible live coral or algae). Surveys and an herbivore exclusion experiment were conducted at all sites, and additional experimental work was performed at a study site (STRI Point) representative of the dominant pattern of coral mortality without macroalgal growth. Survey sites comprised major reefs within Bahia de Almirante and exhibited similar species compositions with varying coral and algal cover. All work was conducted at 5–7 m depth. Research permits were provided by the Autoridad de Recursos Acuático de Panamá and the Autoridad Nacional del Ambiente de Panamá (No. SE/A-48–13).

### Habitat and Herbivore Surveys

We quantified the percent cover of live coral, dead coral, and macroalgae at each site by haphazardly placing twenty 60 × 60 cm quadrats, and counting the proportion of 25 points in each quadrat over a given category. Urchin populations were surveyed by counting all urchins within twenty haphazardly placed 60 × 60 cm quadrats at each site and measuring the test diameter of a representative subset of 50 *E. viridis* urchins. Urchin counts were performed during both day and night, however, since counts were similar in both time periods, only day time survey results are presented here. We then scaled density and size frequency counts of *E. viridis* to biomass using an allometric scaling relationship that we derived from the test diameter and wet biomass of 50 randomly selected *E. viridis* [Biomass (g) = −8.668325 + 0.6678116 * Diameter (mm) + 0.0378074 * (Diameter (mm) − 20.52)^2^]. We measured abundances of all urchin species, but only *E. viridis* had sufficient numbers to warrant biomass estimates.

We quantified the abundance of herbivorous fish by surveying their populations at each of our twelve study sites following a protocol modified from Rotjan and Lewis^55^, which involved identifying, counting, and estimating sizes of herbivorous fish (to 5 cm size classes) along three 15 × 4 m transects at each site. Fish biomass was estimated by converting length data to biomass using the allometric length-weight conversion *W = aL*^*b*^, where parameters *a* and *b* are constants for allometric growth determined from the literature^56^,^57^ and length of individuals was considered as the median value of each size class. We conducted supplemental 80–200 minute visual searches at each site to estimate and compare relative individual body sizes of less abundant herbivores, which were converted to biomass as above (*D. antillarum* biomass estimated from equation in ref. 58).

### Algal Transplant Experiment

We conducted an macroalgal transplant experiment to test the effects of abiotic conditions on algal persistence and the ability of herbivory to limit algal establishment on reefs with extensive dead coral substrate and little standing algal biomass. We transplanted five of the most common types of macroalgae in our study area (*Halimeda* spp., *Dictyota* spp. *Amphiroa* spp., *Lobophora* spp., and *Sargassum* spp.) to the reef at STRI Point. Herbivore access to algae was manipulated with mesh exclusion cages. Thirty replicate plots > 2 m apart were established in a matrix of dead coral. One bundle of each algal type was placed in each plot, with each species averaging *c*7% of the plot area, to mimic the mixed assemblages observed in the field. Each algal species was kept from touching one another to minimize potential allelopathic and associational defense interactions. Plots were randomly assigned to one of three treatments: full cage to exclude herbivores, partial cage to control for potential caging artifacts, and open (no cage) to allow herbivore access. Full (30 × 30 × 15 cm, L × W × H) and partial (control) cages were constructed of aquaculture-grade extruded plastic mesh (mesh size = 12 mm). The roofs of partial cages were suspended 15 cm above the cage wall to allow herbivore access. Both urchins and fish were observed to move freely in and out of partial cages during the experiment. All cages were cleaned as needed to prevent fouling and to remove occasional intruding herbivores from full cages.

To examine changes over time in the relative abundance of a given algal species in each treatment, each plot was photographed at the start of the experiment and at the end of the experiment, 22 days later. A grid with 100 points was overlaid on each photograph using Image J (version 1.46r) and the number of points falling on each macroalgal species was recorded to measure initial and final percent cover and to calculate the change in percent cover over the duration of the trial period. To examine the effect of grazing on biomass, the algae remaining in each plot at the end of the experiment were collected, blotted dry, and weighed. Final wet biomass and percent cover data were analyzed using a split-plot ANOVA with caging treatment as the whole-plot factor and algal type as the sub-plot factor.

### Herbivory x Nutrient Enrichment Experiment

Herbivory and nutrient enrichment are two factors commonly thought to drive coral-algal phase shifts^8^,^27^,^30^. We tested the separate and interactive effects of these factors in limiting algal recruitment and growth using a fully factorial field experiment that crossed three levels of herbivory (full cage, partial cage, and open) and two levels of nutrients (ambient and enriched) using a standardized settlement substrate. Ten replicate tiles (unglazed terracotta tiles, 20 × 20 cm) were randomly assigned to each treatment combination (60 total replicates), and spaced >2 m apart. Tiles were conditioned for 10 days in a flow-through seawater tank at the Bocas del Toro Research Station before being placed in the field. Full cages and partial cages were constructed as described for the above algal transplant experiment. Nutrient enrichment was achieved by suspending a small fiberglass mesh bag (mesh size 1.4 mm) containing 30 g of Osmocote slow-release fertilizer 19-6-12 (N-P-K) above the center of each tile, which was replaced every two weeks. Cages were cleaned as necessary to prevent fouling; however, sixteen of the 60 plots were compromised by the establishment of damselfish (*Stegastes planifrons*) territories which secondarily excluded herbivores and were therefore excluded from the experiment.

After four months, tiles were collected in individual bags and returned to the laboratory for analysis. Tiles were gently rinsed with seawater to remove sediment. Algae were scraped from tiles with a razor blade, blotted dry, and weighed. A two-way ANOVA with caging treatment and nutrient enrichment as fixed factors was used to analyze differences in algal wet biomass (log + 1 transformed to meet ANOVA assumptions).

### Herbivore Size Exclusion Experiment

We experimentally tested the grazing impacts of herbivores of differing body sizes by using size-selective mesh exclusion cages. Access to algae by varying herbivore size classes was manipulated through five caging treatments (jumbo mesh full cage, small mesh full cage, jumbo mesh partial cage, small mesh partial cage, and open plot with no cage). All cages were 30 × 30 × 15 cm and constructed as described above. Jumbo mesh cages (mesh size = 50 mm) excluded entry by large herbivores (e.g., *Diadema* and *Sparisoma viride*), but allowed for the passage of small herbivores including urchins and fish (e.g., *Echinometra* and *S. iseri*). Small mesh cages (mesh size = 12 mm) excluded all herbivore groups listed above, but any mesograzers (e.g., herbivorous snails, amphipods) were able to enter. *Echinometra* and small fish were observed to move freely in and out of jumbo mesh full cages and were never observed in small mesh full cages.

Plots were established in a matrix of dead coral (>2 m apart) randomly assigned to one of the five treatments (n = 10 plots per treatment). Each plot received *c*15 g of *Lobophora* spp., which was chosen due to ease of handling and representative preference by herbivores determined in the algal transplant experiment described above. All cages were cleaned as needed to prevent fouling, however, two full jumbo mesh cages were adopted as territories by aggressive damselfish (*Stegastes planifrons*) so were excluded from the analysis. After 30 days, all remaining algae were collected, blotted dry, and weighed. Final wet biomass was analyzed using a one-way ANOVA with caging treatment as a fixed factor.

### Multi-site Herbivory Exclusion

We examined spatial variation in herbivory pressure by transplanting algae (*Lobophora* spp.) from a common site to all 12 study sites. At each site, we established 20 plots (>2 m apart) assigned to one of two herbivory treatments (full cage and open). Herbivore exclusion cages (25 × 10 × 10 cm) were constructed from aquaculture-grade plastic extruded mesh (mesh size = 7 mm). Approximately 1.5 g of *Lobophora* spp. was placed within each plot. After two days, samples were collected and returned to the laboratory where they were blotted dry and weighed. Biomass data were analyzed using a two-way ANOVA with caging treatment and site as fixed factors (data log + 1 transformed to meet ANOVA assumptions). We examined the relative strength of herbivory at each site by quantifying the percent of algae eaten (herbivory intensity = [(Mean caged biomass-Mean open biomass)/Mean caged biomass]). Sites were considered to have significant herbivory intensity if greater than or equal to 50% of algae present was consumed (herbivory intensity value of 0.5 or higher). A two-sample t-test was then used to examine differences in mean cover of algae (quantified in surveys) between sites with significant and non-significant herbivory. We used linear regressions to investigate the relationships between herbivory intensity and herbivore (urchin and fish) biomass.

## Additional Information

**How to cite this article**: Kuempel, C. D. and Altieri, A. H. The emergent role of small-bodied herbivores in pre-empting phase shifts on degraded coral reefs. *Sci. Rep.* **7**, 39670; doi: 10.1038/srep39670 (2017).

**Publisher's note:** Springer Nature remains neutral with regard to jurisdictional claims in published maps and institutional affiliations.

## Supplementary Material

Supplementary Figure 1
