# Supplementary material for: The emergent role of small-bodied herbivores in pre-empting phase shifts on degraded coral reefs
Source: Sci Rep. 2017 Jan 5;7:39670. doi: 10.1038/srep39670 (PMC5215077; doi:10.1038/srep39670)
Supplement: Supplementary Figure 1 [file srep39670-s1.pdf]

The emergent role of small-bodied herbivores in pre-empting phase shifts on degraded coral reefs

Caitlin D. Kuempel and Andrew H. Altieri

Supplementary information:

Supplementary Figure 1. Map of the Bocas del Toro Archipelago and the twelve sites where surveys and experiments were conducted: Casa Blanca (A), STRI Point (B), Finca (C), Arco Iris (D), Seagal (E), Mystery Spot (F), Punta Rodriguez (G), Cayo Hermanas (H), Pastores Outside (I), Pastores Inside (J), Cayo Roldan (K), Tierra Oscura (L). Map was created using ArcGIS v10.2.2[1].

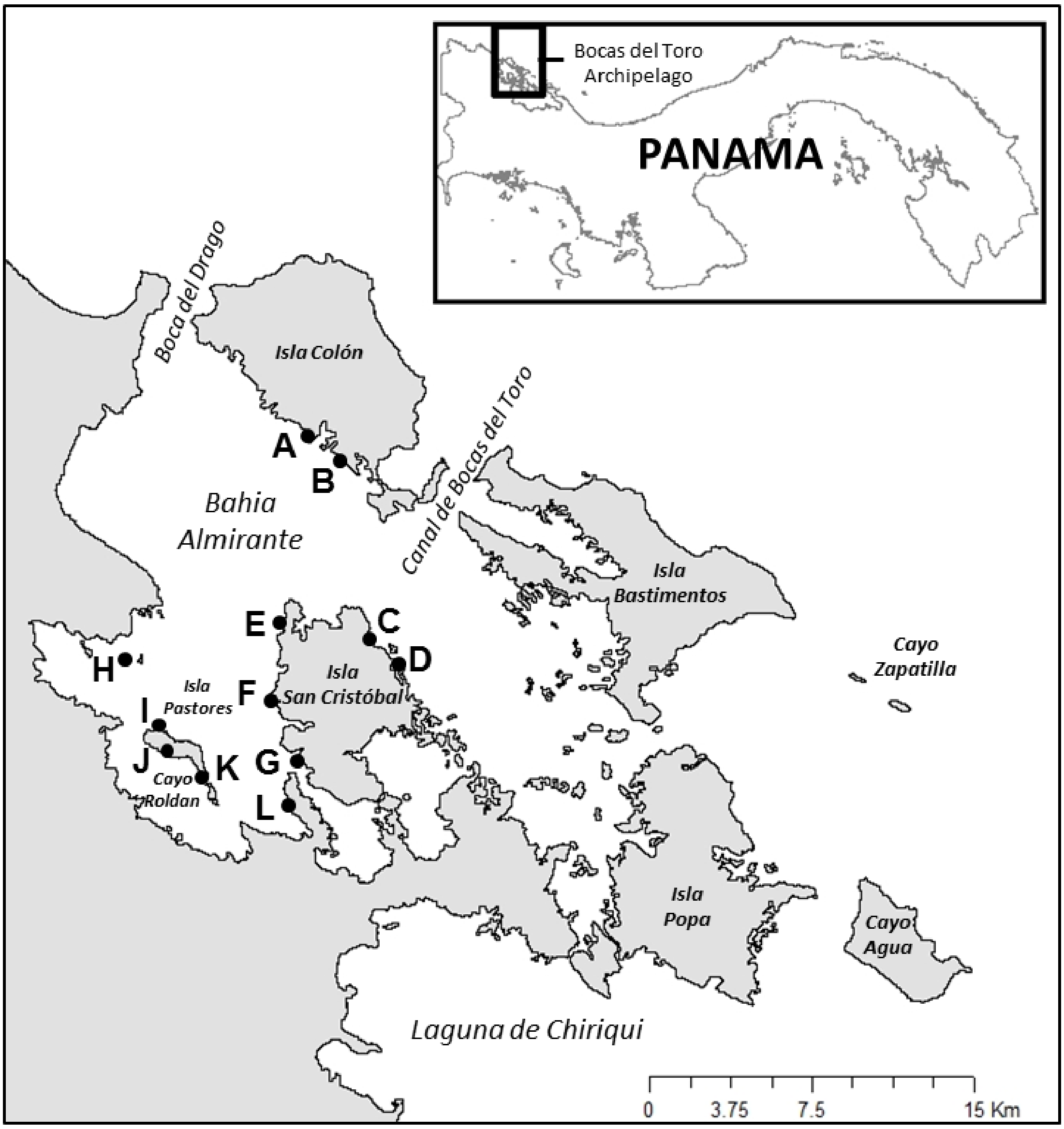

References

1. ESRI. ArcGIS Desktop: Release 10. (2011). at <www.esri.com>
